# Mesoporous Manganese Oxide/Lignin-Derived Carbon for High Performance of Supercapacitor Electrodes

**DOI:** 10.3390/molecules26237104

**Published:** 2021-11-24

**Authors:** Hersandy Dayu Kusuma, Rochmadi, Imam Prasetyo, Teguh Ariyanto

**Affiliations:** 1 Department of Chemical Engineering, Faculty of Engineering, Universitas Gadjah Mada, Jl Grafika No. 2, Yogyakarta 55281, Indonesia; hersandy.d.k@mail.ugm.ac.id (H.D.K.); rochmadi@ugm.ac.id (R.); imampras@ugm.ac.id (I.P.); 2 Carbon Material Research Group, Faculty of Engineering, Universitas Gadjah Mada, Jl Grafika No. 2, Yogyakarta 55281, Indonesia

**Keywords:** lignin, manganese oxide, mesopores carbon, kinetic study, supercapacitor, surfactant

## Abstract

This study explores the modification of lignin with surfactants, which can be used as a template to make mesoporous structures, and can also be used in combination with manganese oxide to produce manganese oxide/lignin-derived carbon. Organosolv extraction, using ethanol (70%) at 150 °C, was carried out to extract lignin from oil palm wood. Lignin was then mixed with Pluronic F-127, with and without Mn(NO_3_)_2_, and then crosslinked with acidic formaldehyde, resulting in a carbon precursor-based modified lignin. Carbonization was carried out at 900 °C to produce lignin-derived carbon and manganese oxide/lignin-derived carbon. The characterization materials included Fourier transform infrared (FTIR) spectroscopy, scanning electron microscope-energy dispersive X-ray (SEM-EDX) mapping, X-ray diffraction (XRD), and N_2_-sorption analysis. FTIR curves displayed the vibration bands of lignin and manganese oxide. SEM images exhibited the different morphological characteristics of carbon from LS120% (lignin with a Pluronic surfactant of 120%) and LS120%Mn20% (lignin with a Pluronic of 120% and Mn oxide of 20%). Carbon LS120% (C-LS120%) showed the highest specific surface area of 1425 m^2^/g with a mean pore size of 3.14 nm. The largest mean pore size of 5.23 nm with a specific surface area of 922 m^2^/g was exhibited by carbon LS120%-Mn20% (C-LS120%-Mn20%). C-LS120%Mn20% features two phases of Mn oxide crystals. The highest specific capacitance of 345 F/g was exhibited by C-LS120%-Mn20%.

## 1. Introduction

Lignin, one of the carbon precursors with the most potential, can be found contained in biomass [1]. Several methods are used to isolate the lignin from biomass, including organosolv [2], kraft [3], and alkali treatment. To obtain a porous carbon, lignin must be treated through pyrolysis under inert conditions or in a vacuum (as part of the carbonization process) [4]. Because of its advantageous properties, porous carbon is an important material that is applied in many subject area, such as energy storage [5], purification [6], adsorption [7], and catalysis [8].

An electric double layer capacitor is a supercapacitor, i.e., an electrical storage device, which can use carbon as an electrode, including lignin-derived carbon. However, previous research has shown that lignin-derived carbon has a low capacitance, which is caused by small pores, low hydrophilicity, and low specific surface area. Improving its electrochemical properties by modifying the characteristics of porous carbon is an on-going concern.

The specific surface area and porosity of carbon have been explored as areas where the electrochemical properties of carbon electrodes can be improved. To improve in these areas, carbon material can be developed using a template. Surfactants are commonly used as a template material because they can modify pore structures. Surfactants are divided into two types: ionic and non-ionic surfactants. The molecular weight of the surfactant plays a role, because the size arrangement of the template is important, as is temperature, concentration, and the solvent used during synthesis. One surfactant that can be used to develop lignin-derived carbon is Pluronic F-127. Pluronic F-127 has a higher molecular weight than other surfactants, and is also one of the materials that can be used as a template for developing mesoporous structures. Furthermore, metal oxide loading can be also performed to improve the electrochemical properties of carbon as an energy storage material [9].

Manganese oxide has good properties: it has good electrical conduction, it is magnetic and catalytic, and it is also environmentally friendly [10]. Because of these properties, it is often applied in many subject areas, especially for energy storage. The crystal phases of manganese oxide consist of MnO, Mn_3_O_4_, Mn_2_O_3_, and MnO_2_ [11]. The high performance of manganese oxide as a supercapacitor has been demonstrated in many studies [12]. However, due to its pseudocapacitive behavior, it results in a low power density with respect to capacitive double layers. Therefore, combining manganese oxide and porous carbon could produce a supercapacitor with an optimized performance.

In this research, composite materials, developed with lignin-derived carbon and manganese oxide/lignin-derived carbon, were synthesized and tested as supercapacitor electrodes. Lignin was extracted from *Elaeis guineensis Jacq* (oil palm wood) using the organosolv method. Manganese oxide was added to lignin-derived carbon to improve the properties of the material through a redox reaction. This work includes not only the characterization and testing of the performance materials as electrodes, but also a kinetic study to investigate the effects of modification with surfactants and manganese oxide on the carbonization process. 

## 2. Results and Discussion

### 2.1. Lignin Extraction

Lignin from *Elaeis guineensis Jacq* (oil palm wood) was extracted using the organosolv process, using ethanol in a single-step fractionation process, resulting in an average yield of 30% (pentaplicate). Using alcohol with a longer alkyl chain (for instance ethanol) leads to a higher yield of a purer product during extraction [13]. During the fractionation process, the solvent breaks the complex *beta aryl ethers* bond (*β-O4*) into its simple components [14]. Increasing the temperature of the extraction process increases the amount of extracted lignin that can be obtained, but reduces the molecular weight of the lignin. 

### 2.2. A Kinetic Study of the Carbonization Process

**Figure 1:**
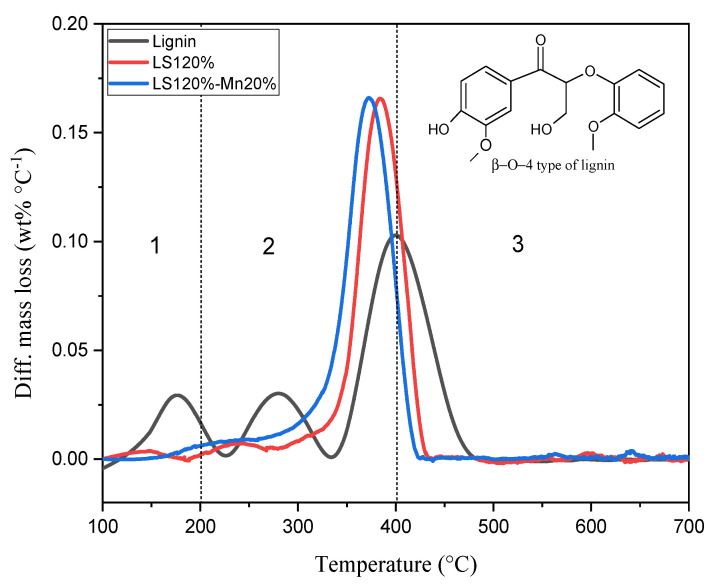
Difference mass loss of lignin, LS120%, and LS120%-Mn20%.

To evaluate thermal behavior during the stages of carbon synthesis, a kinetic study was performed using thermogravimetric analysis. There were two materials for the kinetic study analysis: lignin with a surfactant addition (120%) named LS120% and a lignin+surfactant+Mn precursor (120% surfactant, and 20%Mn) named LS120%-Mn20%. The differential mass loss (DTG) of lignin and the modified materials during thermal decomposition are shown in Figure 1. In general, the degradation process is divided into three stages: the dehydration stage, the fast decomposition stage, and the slow decomposition stage [15]. The dehydration stage occurs in temperatures below 200 °C and is followed by the loss of organic and volatile compounds. Rapid decomposition (active pyrolysis) occurs in the 200–400 °C range, where the material is partially converted into volatile compounds. The slow decomposition stage begins with temperatures above 400 °C [16]. Figure 1 shows that the lignin sample without modification was directly carbonized, with three peaks indicating that the extracted material still contained lignin (340–480 °C), cellulose (230–340 °C) [17], and hemicellulose (150–230 °C). The opposite is seen in materials that do not show peaks of cellulose and hemicellulose. This is possible because there is a polymerization reaction during the templating process. There is an increase and shift in the peak compared to the lignin sample. The presence of the surfactants that were bound to the lignin resulted in a decrease in the decomposition temperature range to 330–420 °C [18]. The slight increase in the DTG curve in the LS and LS-Mn samples was due to the presence of surfactants and formaldehyde, which led to a higher decomposition rate [19]. The presence of manganese oxide caused a peak shift that is not significant, with the peak height of the DTG remaining the same. This is because there is no Mn that decomposes and forms gas or that is carried away by nitrogen flow gas. In the decomposition of lignin, the carboxyl group will tend to decompose into CO_2_ [20], with ether decomposing into CO at low temperatures [14]. The formation of CO from the carbonization process in biomass tends to result from the decomposition of cellulose. Charred material tends to be produced by the demethoxylation of syringyl units [21]. In the carbonization process at 350–400 °C, the C=O-C-CH_2_-OH component will produce two possible types of product. The first product type is a benzene ring, such as guaiacol, and the second product type is two benzene rings. However, as the carbonization temperature increases, the process tends to result in the formation of a benzene ring [15]. The formation of monomers, such as guaiacol, coniferyl alcohol, and species radicals, will induce a polymerization reaction as a side reaction of the decomposition process in the formation of charred material [4].

**Figure 2:**
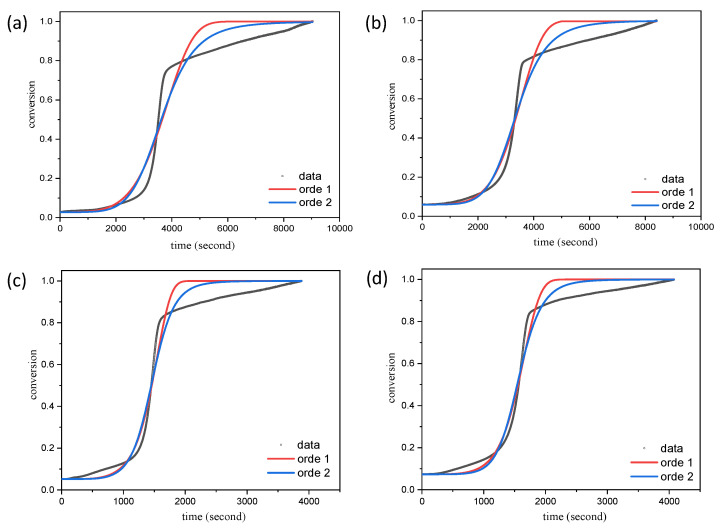
Model fitting for the thermal decomposition kinetic analysis of the material carbonization process: heating rate 5 °C/minutes for (**a**) LS120% and (**b**) LS120%-Mn20%; heating rate 10 °C/min for (**c**) LS120% and (**d**) LS120%-Mn20%.

Thermal decomposition curve analysis was used as the basis for a kinetic study that was performed to investigate the activation energy and pre-exponential factor. The variation of the heating rate (5 and 10 °C/min on a nitrogen gas stream) of the carbonization material was used to identify the effect on the kinetic parameter. The flow of nitrogen gas is used because it can ignore the various factors that arise due to the oxidative atmosphere [22]. Although this does not affect the kinetic parameters, it lowers the yield produced in the carbonization process [17]. Studies related to the decomposition kinetics of LS120% and LMn20% assume that the reaction proceeds at reaction orders of one and two. The use of the free model equation is more suitable for obtaining the values of *Ea* and *A* than other equations, such as the Kissinger equation, because it avoids the discrepancies that occur due to the influence of the multistep reaction [23]. The presence of functional groups will have a significant effect on the overall decomposition reaction and on the distribution of the decomposition products on the equation model [24]. Figure 2a–d shows that the model follows the second-order reaction equation, which is corroborated by the presence of fitted lines that sit close to the data. The value of the activation energy and the pre-exponential factor from the modeling of the LS120% sample and the LS120%-Mn20% sample decomposition processes are shown in Table 1.

The activation energy value during pyrolysis is the minimum energy needed for lignin to become carbon. The value of the activation energy in the carbonization process that involves inert gas will be greater than in atmospheric conditions due to the influence of the presence of oxygen in the environment. The greater the heating rate, the greater the heat transfer, which means that more particles react. Over a certain period, the frequency of particle reaction increases, which is shown by the value of *A*. However, the heating rate does not affect the activation energy value in the material carbonization process. The composite material (LS120%-Mn20%) has a lower activation energy value than LS120%. The lower activation energy value is due to the presence of manganese oxide, which also acts as a catalyst during the carbonization process. The presence of a catalyst helps in lowering the activation energy, which is seen significantly at the heating rate of 10 °C/min for the second-order reaction. In addition to the effect of lowering the activation energy, the presence of a catalyst can also reduce the yield of charred material because it breaks the molecular components of lignin into a gas [25]. The heating rate also causes the mass reduction rate to increase, which affects the mass transfer process. The carbonization process will be less efficient as the heating rate increases due to the poor heat conduction process from the external part of the particle to its internal part [26]. This is quite influential if the material has a high content of volatile compounds [16]. 

### 2.3. Material Characterization

#### 2.3.1. FTIR Spectra

**Figure 3:**
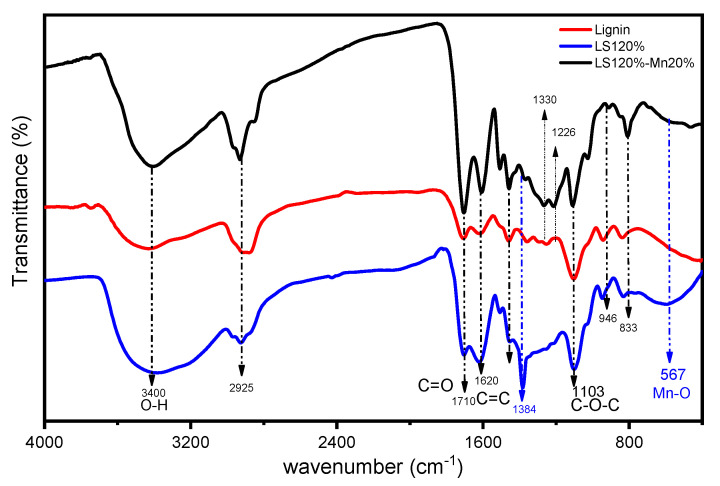
FTIR spectrum of modified and unmodified lignin.

Figure 3 shows the FTIR spectra of lignin during the organosolv extraction compared to the modified lignins. According to the FTIR spectrum, lignin is similar to the LS120% and LS120%-Mn20% compounds. The hydroxyl bond (-OH) in lignin is indicated by a strong and broad peak in the wavenumber at 3400 cm^−1^, while the 2900–2700 cm^−1^ range shows the vibration of the C-H bond in methoxyl from the methyl group [27,28]. The absorption bands at wavenumbers 1710 cm^−1^ and 1620 cm^−1^ show a peak which indicates a stretching vibration of the unconjugated C=O and C=C frame of the benzene aromatic group [1,21,29]. The lignin constituent group is also shown to have bending properties at the 1518 cm^−1^ guacyl (G) component, aromatic rings at the G and S lignin units (1272–1263 cm^−1^ and 1330–1326 cm^−1^) [30], a C-O-C band center position (1025 cm^−1^) [31], and C-O-C vibrations that are influenced by the hydrogen band (1103 cm^−1^). In the FTIR spectrum of lignin, there is a syringyl (S) at 809 cm^−1^. The S/G ratio of lignin represents the lignin fractionation caused by solvent extraction [32]. The composite material of manganese oxide/lignin displays a discrepancy in its FTIR peaks [33]. The key difference between the modified and unmodified material is the existence of the Mn-O tetrahedral vibration band and the Mn-O stretching (567 cm^−1^). The presence of Mn in the composite material causes the surface material to become more polarized by increasing the hydrogen bonds [34].

#### 2.3.2. Surface Morphology and Elemental Contents

**Figure 4:**
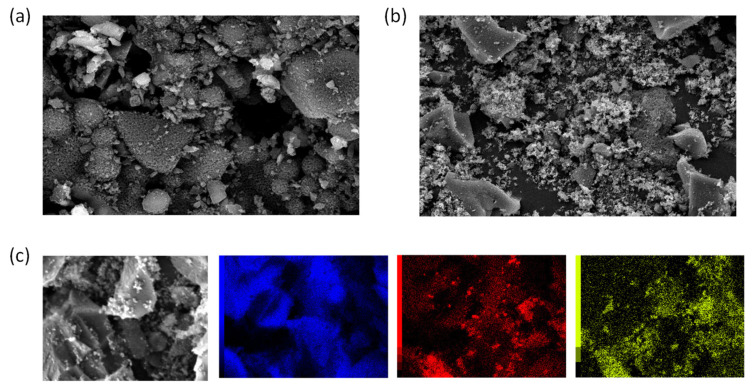
SEM image, magnified 5000×, of: (**a**) C-LS120% and (**b**) C-LS120%-Mn20%. (**c**) SEM mapping of C-LS120%-Mn20%, with coloring of the carbon (blue), oxygen (red), and manganese (green).

The surface and particle morphology of the carbon in C-LS120% and C-LS120%-Mn20% was observed using SEM. Figure 4 displays the C-LS120% particle, which has a more spherical shape than the C-LS120%-Mn20% particle [35]. The spherical shape of the particles is caused by the surfactant, which acts as a template during the synthesis process. Compared to C-LS120%, C-LS120%-Mn20% has ambiguously shaped particles. The presence of Mn in C-LS120%-Mn20% makes the lignin and Mn compete to interact with the surfactant, which leads to the smaller particle sizes of C-LS120%-Mn20%. 

Combined with SEM mapping, energy-dispersive X-ray analysis was used to measure the elemental content in C-LS120%-Mn20% and map its elements. Analyzing these elements is essential to confirm the content in the material (see Table 2). Based on Figure 4c, the SEM image shows that the elements contained in the material composite (C-LS120%-Mn20%) are evenly dispersed. 

#### 2.3.3. Pore Structures

**Figure 5:**
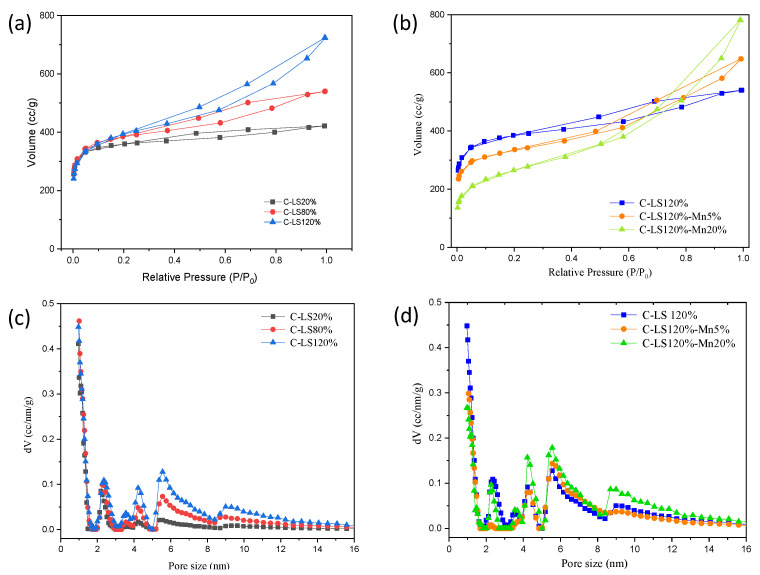
Isotherm curve of nitrogen sorption at STP for (**a**) C-LSy% and (**b**) C-LS120%-Mnx% material, and pore size distribution curve for (**c**) C-LSy% and (**d**) C-LS120%-Mnx% material.

The pore size and specific surface area play an important role in the electrode material for supercapacitors. N_2_ sorption analysis was used to evaluate the pore structures of the materials. Materials C-LS120% and C-LS120%2-Mn20% both have an IV-type isotherm of adsorption and desorption nitrogen (see Figure 5a,b). The curve with an IV-type isotherm in the figure shows a hysteresis loop, which indicates the presence of mesoporous structures in the material [36]. The broadening of the hysteresis loop indicates the domination of mesoporous structures [37]. The variation of surfactants shows that hysteresis increases with an increase in surfactant content. 

Figure 5c,d show the pore size distribution of the carbon material. With a variety of surfactants, the addition of more surfactants caused an increase in the mesoporous structure of the materials used in this study [38]. This is also similar to the increase in the amount of manganese oxide. Figure 5d shows that the C-LS120%-Mn20% sample experienced a decrease in micropore volume with an increase in the mesoporous volume when compared to the C-LS120% and C-LS120%-Mn20% samples. During the carbonization process, several components, including surfactants, play a role in forming the pore structure which will affect the pore volume of the material. Micropores are predominantly produced due to the flow of steam [39], while mesopores are caused by the decomposition of surfactants as they act as templates [40]. The decrease in micropore volume is also caused by the presence of manganese, which makes the pores micro sized when the carbonization process is widened [41]. This is because the catalytic process that occurs due to the presence of metal oxides decomposes lignin into H_2_, CO, CO_2_, or CH_4_ [25,42,43]. The higher carbonization temperature could widen the pore size in the catalytic carbon material [35]. The microporous structure that is formed from pyrolysis due to steam at a temperature of 900 °C has a size range of 0.8–1.9 nm [39]. The material that has the addition of a surfactant has a larger specific surface area than the material that has the addition of manganese oxide, as seen in Table 3. The largest surface area was caused by the addition of 120% wt surfactant in the lignin. Although the addition of the surfactant to C-LS120% and C-LS80% did not have a large significant effect on the surface area, it did have a significant effect on the micropore volume and the average pore diameter, as summarized in Table 3.

#### 2.3.4. Crystal Phase

**Figure 6:**
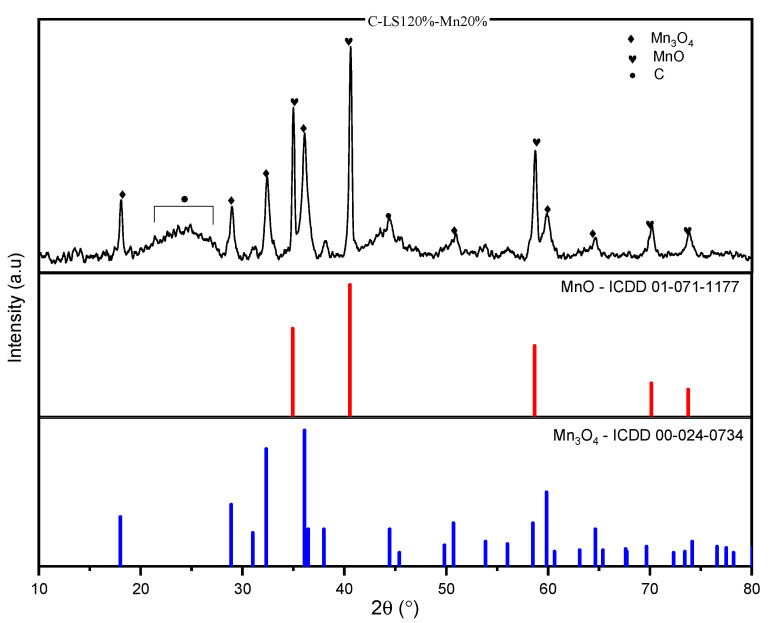
XRD pattern of C-LS120%-Mn20% compared with ICDD reference data for Mn_3_O_4_ and MnO.

Manganese oxide crystal phases (Mn_x_O_y_) were evaluated using X-ray diffraction (XRD) in a 2*θ* range of 10–80° [44]. Figure 6 shows that C-LS120%-Mn20% has two phases of its crystal structure, one containing Mn_3_O_4_ and the other MnO. The analysis was performed considering the peaks at 2*θ* in 29°, 32° dan 36° [45] for Mn_3_O_4_ and 34°, 40° dan 58° [46,47,48] for MnO, which is compared to database ICDD card numbers, 00-024-0734 and 01-071-1177.

The presence of two phases is caused by the presence of Mn, temperature, and oxygen during the carbonization process of the material [49]. MnO is the result of a reduction from Mn_3_O_4_. During the carbonization process, the Mn_3_O_4_ was reduced, becoming MnO at temperatures of 600 °C [50]. Two phases of crystals in C-LS120%-Mn20%, 2*θ* in 20–28° and 42–47°, show the humped peaks [51]. The humped peaks are caused by the properties of the carbon present in the material.

### 2.4. Electrochemical Properties

**Figure 7:**
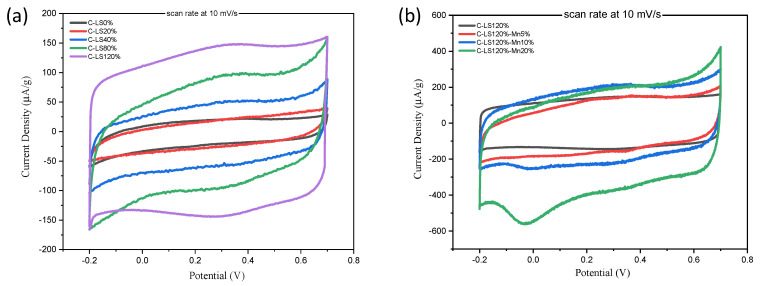
Cyclic voltammetry curve at a scan rate of 10 mV/s for: (**a**) surfactant variations; (**b**) manganese oxide variations.

Cyclic voltammetry (CV) curves of the synthesized materials are shown in Figure 7a for various surfactant additions and Figure 7b for various manganese oxide additions. Porous carbon with a 10% Mn loading was prepared and tested to examine the electrochemical performance of the material against loadings of between 5% and 20%. It was expected that the characteristics would be in between those of the 5%- and 20%-loaded material. All materials were pre-treated with hydrogen peroxide to increase the surface wettability [52]. The CV curves of the surfactant variation have a quasi-rectangular shape because the electrochemical process is dominated by ion adsorption on the surface of the material [53].

The scanning rate for the C-LS120% variation, shown in Figure 7a, indicates the presence of a hump at a voltage of 0.18–0.25 V due to the contribution of pseudo-capacitance caused by the presence of oxygen on the carbon surface [54]. The increase in the amount of oxygen on the carbon surface increases the capacitance of the material. Compared to C-LS120%, the material of C-LS120%-Mnx (the variation with manganese oxide) shows a peak in the voltage range of −0.1 to 0.1 V [55,56] that is evidence of the redox process at a low scanning rate. The crystal phase affected the specific capacitance due to the ion transfer and the change in the oxidation state of the manganese oxide. The crystal structure induced a surface charge in the initial step of oxidation. It indicated the involvement of electrolyte ion intercalation in the network of structures, which is accommodated at the oxygen-vacant site that strongly interacts with Mn atoms [57]. The crystal phase structure of manganese shows the distribution of a number of oxygen and manganese sites, and both of them are important to the electrochemical performance, especially as it relates to the specific capacitance. The pure Mn_3_O_4_ has poor conductivity and cyclic stability as an electrode [58]. However, it could be improved by making Mn_3_O_4_ into a composite with carbon [59]. The porous manganese oxide–carbon composite increases conductivity and has excellent specific capacitance and cyclic stability [58,59]. The mechanism of the discharging reaction in composite materials is estimated to follow Equation (1) [56].

Mn(III)_2_Mn(II)O_4_ + xC^+^ + yH^+^ + (x + y)^e−^ ↔ Mn(III)_(2−(x+y))_ Mn(II)_(1+x+y)_O_4_C_x_H_y_ (1)

The C-LS120% sample has the largest capacitance among the surfactant variations in this study. This is due to the influence of the specific surface area, which is greater than the other variations (although the specific surface areas of C-LS120% and C-LS80% are not much different). Table 3 shows that the average pore size of the samples is different, so the pore structure will affect the electrolyte ion transfer process [60]. The specific capacitance value is also influenced by the scan rate, which is related to the ion transfer process and ion contact with the material. This phenomenon occurs due to the diffusion of ions from the bulk electrolyte to the diffusion surface and the possibility of the intercalation of ions into the crystal host structure [61]. For the composite material of C-LS120%-Mn, all variations show a high value of specific capacitance due to the additional redox charge. The highest specific capacitance of 345 F/g is featured by C-LS120%-Mn20%, which is superior to the other studies listed in Table 4. It is important to mention that further studies are needed to characterize the electrochemical properties through electrochemical impedance spectroscopy (EIS), the Nquist plot, and charge/discharge and cycle life measurement.

## 3. Materials and Methods

### 3.1. Material 

The materials used were *Elaeis guineensis Jacq* (oil palm wood) as the lignin-derived carbon source, ethanol, methanol triblock copolymer surfactant (Pluronic F-127, Sigma Aldrich, Singapore), Mn(NO_3_)_2_·4H_2_O from Merck Germany, formaldehyde solution 37% from Merck Germany, hydrogen peroxide solution 15%, Nafion (5% purity, Sigma Aldrich), isopropyl alcohol (99% purity, Merck, Darmstadt, Germany), hydrogen peroxide (50% purity, PT Peroksida Indonesia Pratama, Karawang, Indonesia), and aquadest.

### 3.2. Preparation

#### 3.2.1. Lignin Extraction

Lignin was extracted from oil palm wood (OPW) using organic solvents (ethanol 70% *v*/*v*) in a single-step fractionation process. OPW was crushed and ground under a 50 mesh and mixed with ethanol in an autoclave with a temperature of 150 °C for 150 min and under nitrogen pressure. The black liquor was separated from the solid phase of the extract, then added with aquadest to pH 2 and precipitated. The precipitated result was washed using aquadest to neutral and filtered, followed by drying in the oven at 105 °C.

#### 3.2.2. Porous Carbon and Composite Synthesis

Mesoporous carbon material was prepared by mixing precursors. The lignin from the extraction was used as a carbon source. Four grams of lignin were mixed with the surfactant, Pluronic F-127, at variations of 20%, 40%, 80%, and 120% wt (denoted as “y%”) of lignin. They were put into 30 mL distillate water, which contained 3 mL of 1 M HCl and 5 mL of 37% formaldehyde. The mixture was stirred overnight so that it would completely react. The sample was poured into a Petri dish, dried, and then carbonized in an N_2_ atmosphere, and then activated using steam at a temperature of 900 °C for 2 h. For the composite material, the ratio of lignin and surfactant used was 120 wt.%, while the Mn(NO_3_)_2_·4H_2_O content was varied to obtain 5, 10, and 20 wt.% Mn (denoted as “x%”) in porous carbon. The material and LS120%-Mnx% that were the result of the carbonization were surface oxidized using 15% *v*/*v* of H_2_O_2_ and named C-LSy% and C-LS120%-Mnx%. Detail material preparation is given in reference [64].

### 3.3. Kinetic Study

The study of the carbonization process of the modified material was carried out by thermogravimetric analysis at 700 °C with a heating rate of 5 °C/m and 10 °C/m in a nitrogen atmosphere. It was compared with a model of a kinetic study to determine the activation energy and pre-exponential factor. The irreversible of carbonization lignin was defined as:

(2) $$\mathrm{Lignin}\;\text{organosolv-surfactant}\;\overset{k}{\to }\;\mathrm{carbon}+\mathrm{tar}+\mathrm{syngas}$$

(3) $$\mathrm{Lignin}\;\text{organosolv-surfactant-manganese}\;\mathrm{oxide}\;\overset{k}{\to }\;\text{carbon-manganese}\;\mathrm{oxide}+\mathrm{tar}+\mathrm{syngas}$$

where *k* is defined as the rate constant for a reaction that is temperature dependent:

(4) $$k=A\;e^{\frac{-Ea}{RT}}$$

where *Ea* is the activation energy (kJ/mol), *A* is the pre-exponential factor (minutes^−1^), *T* is the absolute temperature (K), and *R* is gas constant (8.314 J/mol·K). The transformation rate of the solid phase to the volatile phase of the material follows Equation (5):

(5) $$\frac{d\alpha }{dt}=k\left(T\right)f\left(\alpha \right)$$

where *α*, *t*, *k*(*T*), and *f*(*α*) represent the degree of conversion of the process, the time, the rate constant, and the reaction model, respectively. The conversion was normalized from the weight data, which is caused by the decomposition process as follows:

(6) $$\alpha =\frac{\left(m_{i}-m_{t}\right)}{\left(m_{i}-m_{f}\right)}$$

where *m_i_* is the initial mass of the sample, *m_t_* is the actual mass at a certain time, and *m_f_* is the final mass after the carbonization process. Hence, many authors restrict the mathematical function f(α) as:

(7) $$f\left(\alpha \right)={\left(1-\alpha \right)}^{n}$$

where *n* is the reaction order. By substituting Equations (3) and (5), and combining the non-isothermal experiment data with the linear heating rate to Equation (4), the final equation can be written as:

(8) $$\frac{d\alpha }{dT}=\frac{A}{\beta }\cdot {\left(1-\alpha \right)}^{n}\cdot e^{-Ea/RT}$$

### 3.4. Material Characterization

Synthesized materials were characterized using the Fourier transform infrared (FTIR) Thermo Scientific Nicolet iS10 to identify the functional groups of lignin, lignin + surfactant and lignin + surfactant + Mn. FTIR spectra were recorded at a wavenumber range of 4000–400 cm^−1^. The morphological material was characterized using a scanning electron microscope energy dispersion X-ray (SEM-EDX) JEOL JSM-6510LA instrument at a voltage of 15 kV. Analysis of the crystal phase of the composite material was conducted on a Bruker D2 Phaser at 2*θ* range 10–80 using Cu *kα* radiation. The pores and surface area of the materials were analyzed through N_2_ sorption analysis using a NOVA 2000 analyzer (Quantachrome Inc., Boynton Beach, FL, USA).

### 3.5. Material Testing

The material was ground in a mortar and sieved using a 300 mesh and oxidized using 15% H_2_O_2_ with a ratio of material to hydrogen peroxide of 1:200 *w*/*v*. The oxidized material was dried at room temperature. Ten milligrams of sieved materials were dispersed into 1 mL of isopropyl alcohol, which was added to a 20 μL Nafion binder. It was ultrasonicated for 30 min. Ten liters of ink were dropped onto a glassy carbon electrode of 0.3 mm. The materials were tested using a three-electrodes system for measuring capacitance. The materials were mixed with a binder and isopropyl alcohol. Three electrodes (working, counter (Pt), and references (Ag/AgCl)) were dipped into 1 M of H_2_SO_4_ to create an electrolyte solution. Electrochemical testing was carried out using a DROPSENS Stat 400 connected to DROPVIEW 8400 at the potential range of −0.2 to 0.7 V.

## 4. Conclusions

The potential preparation of mesoporous lignin-derived carbon and manganese oxide/lignin-derived carbon has been evaluated. Lignin was successfully extracted from oil palm wood and was modified using surfactants, with and without the addition of Mn oxide precursors. The kinetic model of carbonization followed the second-order equation. The modification of lignin with surfactants and its loading with Mn oxide precursor was successfully used to prepare mesoporous lignin-derived carbon and manganese oxide/lignin-derived carbon. The material characterizations with FTIR, SEM-EDX-mapping, XRD, N_2_-sorption analysis confirmed the success of the material preparation and was used to understand the performance of its electrochemical properties (specific capacitance). The excellent specific capacitance of up to 345 F/g was shown by the carbon material that was prepared through the carbonization of lignin and modified by 120% of surfactant and 20 wt.% of manganese oxides.

## Figures and Tables

**Table 1:** Kinetic parameters of the material carbonization process.

Parameters	Ramp Rate (°C/min)	Order	*A* (/s)	*Ea* (kJ/mol)	*R* ^2^
LS120%	5	1	0.33 ± 0.06	5.00 ± 0.09	0.9730
		2	13.99 ± 2.31	6.70 ± 0.87	0.9832
	10	1	(4.45 ± 1.98) × 10^4^	10.02 ± 0.02	0.9835
		2	(5.22 ± 2.25) × 10^6^	100.25 ± 0.02	0.9878
LS120%-Mn20%	5	1	1.83 ± 0.35	5.59 ± 0.09	0.9786
		2	42.68 ± 7.14	6.95 ± 0.08	0.9866
	10	1	(1.54 ± 0.47) × 10^3^	8.143 ± 0.15	0.9887
		2	(3.40 ± 1.09) × 10^5^	10.07 ± 0.02	0.9915

**Table 2:** Elemental percentages.

Material	Elements (%)		
	C	O	Mn
C-LS120%-Mn20%	74.61	9.25	16.14

**Table 3:** Material structure characteristic.

Materials	Addition of Surfactant/Mn (%.wt)	Pore Characteristics		
		*SSA* * (m^2^/gr)	*V_mic_* ** (%)	*D_ave_* *** (nm)
C-LS	20	1085	79.26	1.89
	80	1411	50.38	2.36
	120	1425	38.06	3.14
C-LS120%-Mn	5	1210	37.26	3.31
	20	922	12.67	5.23

* *SSA*: specific surface area; ** *V_mic_*: micropore volume; *** *D_ave_*: average diameter.

**Table 4:** The specific capacitance of the materials.

Material	Specific Capacitance (F/gr)	Reference
C-LS0%	22.0	This research
C-LS20%	14.1	
C-LS40%	33.6	
C-LS80%	81.7	
C-LS120%	140.9	
C-LS120%-Mn5%	128.3	
C-LS120%-Mn10%	223.8	
C-LS120%-Mn20%	345.3	
Lignin-derived mesoporous carbon	91.7	[40]
MnO/carbon nanofiber	301.8	[56]
Porous carbon/MnO nanosheet	162.7	[47]
Nitrogen-doped porous hollow carbon spheres/MnO_2_	255	[62]
Puffedrice-based biomass carbon	117.2	[63]

## Data Availability

Not applicable.

## Sample Availability

Samples of the compounds of lignin-derived carbon and Mn oxide/lignin-derived carbon are available from the authors.

## References

[1] Zhang P., Dong S.J., Ma H.H., Zhang B.X., Wang Y.F., Hu X.M. (2015). Fractionation of corn stover into cellulose, hemicellulose and lignin using a series of ionic liquids. Ind. Crops Prod..

[2] Prasetyo I., Permatasari P.R., Laksmana W.T. (2020). Lignin Refinery Using Organosolv Process for Nanoporous Carbon Synthesis. Molecules.

[3] Tian D., Chandra R.P., Lee J.S., Lu C., Saddler J.N. (2017). A comparison of various lignin-extraction methods to enhance the accessibility and ease of enzymatic hydrolysis of the cellulosic component of steam-pretreated poplar. Biotechnol. Biofuels.

[4] Kawamoto H. (2017). Lignin pyrolysis reactions. J. Wood Sci..

[5] Zeiger M., Ariyanto T., Krüner B., Peter N.J., Fleischmann S., Etzold B.J.M., Presser V. (2016). Vanadium pentoxide/carbide-derived carbon core-shell hybrid particles for high performance electrochemical energy storage. J. Mater. Chem. A.

[6] Ariyanto T., Masruroh K., Pambayun G.Y.S., Mukti N.I.F., Cahyono R.B., Prasetya A., Prasetyo I. (2021). Improving the Separation of CO_2_/CH_4_ Using Impregnation of Deep Eutectic Solvents on Porous Carbon. ACS Omega.

[7] Hernando A., Ariyanto T., Prasetyo I. (2019). Preserving climacteric fruits by ripening hormone oxidation using nano-KMnO_4_ confined within nanoporous carbon. ASEAN J. Chem. Eng..

[8] Gläsel J., Diao J., Feng Z., Hilgart M., Wolker T., Su D.S., Etzold B.J.M. (2015). Mesoporous and Graphitic Carbide-Derived Carbons as Selective and Stable Catalysts for the Dehydrogenation Reaction. Chem. Mater..

[9] Konnerth P., Jung D., Straten J.W., Raffelt K., Kruse A. (2021). Metal oxide-doped activated carbons from bakery waste and coffee grounds for application in supercapacitors. Mater. Sci. Energy Technol..

[10] Wang K., Zhao K., Wang Y., Li H., Jiang H., Chen L. (2021). N,S co-doped carbon confined MnO/MnS heterostructures derived from a one-step pyrolysis of Mn-methionine frameworks for advanced lithium storage. J. Alloys Compd..

[11] Zang X.-B., Li L.-T., Sun Z.-X., Boukherroub R., Meng J.-X., Cai K.-P., Shao Q.-G., Cao N. (2020). A simple physical mixing method for MnO_2_/MnO nanocomposites with superior Zn^2+^ storage performance. Trans. Nonferr. Met. Soc. China.

[12] Zhou X., Meng T., Yi F., Shu D., Li Z., Zeng Q., Gao A., Zhu Z. (2021). Supramolecular assisted fabrication of Mn_3_O_4_ anchored nitrogen-doped reduced graphene oxide and its distinctive electrochemical activation process during supercapacitive study. Electrochim. Acta.

[13] Zijlstra D.S., Lahive C.W., Analbers C.A., Figueirêdo M.B., Wang Z., Lancefield C.S., Deuss P.J. (2020). Mild Organosolv lignin extraction with alcohols: The importance of benzylic alkoxylation. ACS Sustain. Chem. Eng..

[14] Hosseinaei O., Harper D.P., Bozell J.J., Rials T.G. (2016). Role of Physicochemical Structure of Organosolv Hardwood and Herbaceous Lignins on Carbon Fiber Performance. ACS Sustain. Chem. Eng..

[15] Jiang W., Wu S., Lucia L.A., Chu J. (2017). A comparison of the pyrolysis behavior of selected Β-O-4 type lignin model compounds. J. Anal. Appl. Pyrolysis.

[16] Fernandez A., Saffe A., Pereyra R., Mazza G., Rodriguez R. (2016). Kinetic study of regional agro-industrial wastes pyrolysis using non-isothermal TGA analysis. Appl. Therm. Eng..

[17] Anca-Couce A. (2016). Reaction mechanisms and multi-scale modelling of lignocellulosic biomass pyrolysis. Prog. Energy Combust. Sci..

[18] Jang B., Yang K., Quan B., Piao Y. (2013). Simple synthesis of thin-layered hollow carbon nanostructures by the direct pyrolysis of surfactants. Mater. Lett..

[19] Chen H., Xu G., Xiao C., Bi Y., Hu J. (2019). Fast Pyrolysis of Organosolv Lignin: Effect of Adding Stabilization Reagents to the Extraction Process. Energy Fuels.

[20] Chen Y., Fang Y., Yang H., Xin S., Zhang X., Wang X., Chen H. (2019). Effect of volatiles interaction during pyrolysis of cellulose, hemicellulose, and lignin at different temperatures. Fuel.

[21] Dai G., Zou Q., Wang S., Zhao Y., Zhu L., Huang Q. (2018). Effect of Torrefaction on the Structure and Pyrolysis Behavior of Lignin. Energy Fuels.

[22] Bach Q.V., Trinh T.N., Tran K.Q., Thi N.B.D. (2017). Pyrolysis characteristics and kinetics of biomass torrefied in various atmospheres. Energy Convers. Manag..

[23] Slopiecka K., Bartocci P., Fantozzi F. (2012). Thermogravimetric analysis and kinetic study of poplar wood pyrolysis. Appl. Energy.

[24] Asatryan R., Hudzik J.M., Bozzelli J.W., Khachatryan L., Ruckenstein E. (2019). OH-Initiated Reactions of p-Coumaryl Alcohol Relevant to the Lignin Pyrolysis. Part I. Potential Energy Surface Analysis. J. Phys. Chem. A.

[25] Cui Y., Wang W., Chang J. (2019). Study on the product characteristics of pyrolysis lignin with calcium salt additives. Materials.

[26] El-Sayed S.A., Khairy M. (2015). Effect of heating rate on the chemical kinetics of different biomass pyrolysis materials. Biofuels.

[27] Hu G., Cateto C., Pu Y., Samuel R., Ragauskas A.J. (2012). Structural characterization of switchgrass lignin after ethanol organosolv pretreatment. Energy Fuels.

[28] Ponnuchamy V., Gordobil O., Diaz R.H., Sandak A., Sandak J. (2021). Fractionation of lignin using organic solvents: A combined experimental and theoretical study. Int. J. Biol. Macromol..

[29] Yáñez-S M., Matsuhiro B., Nuñez C., Pan S., Hubbell C.A., Sannigrahi P., Ragauskas A.J. (2014). Physicochemical characterization of ethanol organosolv lignin (EOL) from Eucalyptus globulus: Effect of extraction conditions on the molecular structure. Polym. Degrad. Stab..

[30] Sun Y.C., Wang M., Sun R.C. (2015). Toward an understanding of inhomogeneities in structure of lignin in green solvents biorefinery. Part 1: Fractionation and characterization of lignin. ACS Sustain. Chem. Eng..

[31] Sivasankarapillai G., Eslami E., Laborie M.P. (2019). Potential of organosolv lignin based materials in pressure sensitive adhesive applications. ACS Sustain. Chem. Eng..

[32] Tagami A., Gioia C., Lauberts M., Budnyak T., Moriana R., Lindström M.E., Sevastyanova O. (2018). Solvent fractionation of softwood and hardwood kraft lignins for more efficient uses: Compositional, structural, thermal, antioxidant and adsorption properties. Ind. Crops Prod..

[33] Günay M., Baykal A., Toprak M.S., Sözeri H. (2012). A green chemical synthesis and characterization of Mn_3_O_4_ nanoparticles. J. Supercond. Nov. Magn..

[34] Cui W.G., Zhuang X.Y., Li Y.T., Zhang H., Dai J.J., Zhou L., Hu Z., Hu T.L. (2021). Engineering Co/MnO heterointerface inside porous graphitic carbon for boosting the low-temperature CO_2_methanation. Appl. Catal. B Environ..

[35] Vo T.K., Kim J. (2020). Facile synthesis of mesoporous Cr_2_O_3_ microspheres by spray pyrolysis and their photocatalytic activity: Effects of surfactant and pyrolysis temperature. Korean J. Chem. Eng..

[36] Salim N.V., Mateti S., Cizek P., Hameed N., Parameswaranpillai J., Fox B. (2019). Large, Mesoporous Carbon Nanoparticles with Tunable Architectures for Energy Storage. ACS Appl. Nano Mater..

[37] Ran F., Yang X., Xu X., Li S., Liu Y., Shao L. (2021). Green Activation of Sustainable Resources to Synthesize Nitrogen-doped Oxygen-riched Porous Carbon Nanosheets towards High-performance Supercapacitor. Chem. Eng. J..

[38] Soneda Y. (2013). Carbons for Supercapacitors.

[39] Fu J., Zhang J., Jin C., Wang Z., Wang T., Cheng X., Ma C. (2020). Effects of temperature, oxygen and steam on pore structure characteristics of coconut husk activated carbon powders prepared by one-step rapid pyrolysis activation process. Bioresour. Technol..

[40] Saha D., Li Y., Bi Z., Chen J., Keum J.K., Hensley D.K., Grappe H.A., Meyer H.M., Dai S., Paranthaman M.P. (2014). Studies on supercapacitor electrode material from activated lignin-derived mesoporous carbon. Langmuir.

[41] Byun J.S., Jeong Y.C., Kim J.H., Shin M.C., Park J.Y., Jin H.J., Park C.R., Kim T., Yang S.J. (2021). Pseudo metal-organic coordination derived one-step carbonization of non-carbonizable carboxylate organic molecules toward functional mesostructured porous carbons. Carbon N. Y..

[42] Olcese R.N., Francois J., Bettahar M.M., Petitjean D., Dufour A. (2013). Hydrodeoxygenation of guaiacol, a surrogate of lignin pyrolysis vapors, over iron based catalysts: Kinetics and modeling of the lignin to aromatics integrated process. Energy Fuels.

[43] Navarro-Suárez A.M., Saurel D., Sánchez-Fontecoba P., Castillo-Martínez E., Carretero-González J., Rojo T. (2018). Temperature effect on the synthesis of lignin-derived carbons for electrochemical energy storage applications. J. Power Sources.

[44] Li L.-W., Wang L.-P., Zhang M.-Y., Huang Q.-Z., He K.-J., Wu F.-X. (2020). Enhancement of lithium storage capacity and rate performance of Se-modified MnO/Mn_3_O_4_ hybrid anode material via pseudocapacitive behavior. Trans. Nonferr. Met. Soc. China.

[45] Tian Z.Y., Mountapmbeme Kouotou P., Bahlawane N., Tchoua Ngamou P.H. (2013). Synthesis of the catalytically active Mn_3_O_4_ spinel and its thermal properties. J. Phys. Chem. C.

[46] Li G., Li Z., Hou Z., Liu Y., Jiao S. (2020). Unraveling superior lithium storage performance of MnO by a three-dimensional structure-memory anode. Electrochim. Acta.

[47] Zhao N., Deng L., Luo D., Zhang P. (2020). One-step fabrication of biomass-derived hierarchically porous carbon/MnO nanosheets composites for symmetric hybrid supercapacitor. Appl. Surf. Sci..

[48] Gao M., Dong X., Wang K., Duan W., Sun X., Zhu C., Wang W. (2021). Laser direct preparation and processing of graphene/MnO nanocomposite electrodes for microsupercapacitors. J. Energy Storage.

[49] Dessie Y., Tadesse S., Eswaramoorthy R., Abebe B. (2019). Recent developments in manganese oxide based nanomaterials with oxygen reduction reaction functionalities for energy conversion and storage applications: A review. J. Sci. Adv. Mater. Devices.

[50] Dey S., Praveen Kumar V.V. (2020). The performance of highly active manganese oxide catalysts for ambient conditions carbon monoxide oxidation. Curr. Res. Green Sustain. Chem..

[51] Nam K.M., Kim Y.I., Jo Y., Lee S.M., Kim B.G., Choi R., Choi S.I., Song H., Park J.T. (2012). New crystal structure: Synthesis and characterization of hexagonal wurtzite MnO. J. Am. Chem. Soc..

[52] He Y., Zhang Y., Li X., Lv Z., Wang X., Liu Z., Huang X. (2018). Capacitive mechanism of oxygen functional groups on carbon surface in supercapacitors. Electrochim. Acta.

[53] Zhang X.Q., Lu A.H., Sun Q., Yu X.F., Chen J.Y., Li W.C. (2018). Unconventional Synthesis of Large Pore Ordered Mesoporous Carbon Nanospheres for Ionic Liquid-Based Supercapacitors. ACS Appl. Energy Mater..

[54] Tian J., Wu S., Yin X., Wu W. (2019). Novel preparation of hydrophilic graphene/graphene oxide nanosheets for supercapacitor electrode. Appl. Surf. Sci..

[55] Andreas H.A., Black J.M., Oickle A.A. (2014). Self-discharge in manganese oxide electrochemical capacitor electrodes in aqueous electrolytes with comparisons to faradaic and charge redistribution models. Electrochim. Acta.

[56] Xu W., Liu L., Weng W. (2021). High-performance supercapacitor based on MnO/carbon nanofiber composite in extended potential windows. Electrochim. Acta.

[57] Xu S., Wang T.H., Wang C.F., Chen C.W., Dong C.D., Huang C.P. (2019). The effect of crystal phase of manganese oxide on the capacitive deionization of simple electrolytes. Sci. Total Environ..

[58] Ubale A.U., Waghmare M.A., Iqbal K.S., Pathan H.M. (2020). Manganese oxides: Promising electrode materials for Li-ion batteries and supercapacitors. J. Mater. Sci. Mater. Electron..

[59] Zhang S.W., Chen G.Z. (2008). Manganese oxide based materials for supercapacitors. Energy Mater. Mater. Sci. Eng. Energy Syst..

[60] Zhu Y., Hu H., Li W., Zhang X. (2007). Resorcinol-formaldehyde based porous carbon as an electrode material for supercapacitors. Carbon N. Y..

[61] Nikitina V.A. (2020). Charge transfer processes in the course of metal-ion electrochemical intercalation. Curr. Opin. Electrochem..

[62] Du W., Wang X., Zhan J., Sun X., Kang L., Jiang F., Zhang X., Shao Q., Dong M., Liu H. (2019). Biological cell template synthesis of nitrogen-doped porous hollow carbon spheres/MnO_2_ composites for high-performance asymmetric supercapacitors. Electrochim. Acta.

[63] Xie X.B., Zhang B., Wang Q., Zhao X., Wu D., Wu H., Sun X., Hou C., Yang X., Yu R. (2021). Efficient microwave absorber and supercapacitors derived from puffed-rice-based biomass carbon: Effects of activating temperature. J. Colloid Interface Sci..

[64] Kusuma H.D. (2021). Modifikasi Karbon dari Lignin Menggunakan Oksida Mangan untuk Meningkatkan Performa Elektroda Superkapasitor. Master’s Thesis.

